# The Role of the IL-20 Subfamily in Glaucoma

**DOI:** 10.1155/2016/4083735

**Published:** 2016-01-20

**Authors:** Mary K. Wirtz, Kate E. Keller

**Affiliations:** Casey Eye Institute, Oregon Health & Science University, 3181 S.W. Sam Jackson Park Road, Portland, OR 97239, USA

## Abstract

Glaucoma is a common disease that leads to loss of peripheral vision and, if left untreated, ultimately to blindness. While the exact cause(s) of glaucoma is still unknown, two leading risk factors are age and elevated intraocular pressure. Several studies suggest a possible link between glaucoma and inflammation in humans and animal models. In particular, our lab recently identified a T104M mutation in IL-20 receptor-B (*IL-20RB*) in primary open angle glaucoma patients from a large pedigree. Several of the interleukin- (IL-) 20 family of cytokines and receptors are expressed in ocular tissues including the trabecular meshwork, optic nerve head, and retinal ganglion cells. The DBA/2J mouse develops high intraocular pressures with age and has characteristic optic nerve defects that make it a useful glaucoma model. IL-24 expression is significantly upregulated in the retina of these mice, while IL-20RA expression in the optic nerve is downregulated following pressure-induced damage. The identification of a mutation in the* IL-20RB* gene in a glaucoma pedigree and changes in expression levels of IL-20 family members in the DBA/2J mouse suggest that disruption of normal IL-20 signaling in the eye may contribute to degenerative processes associated with glaucoma.

## 1. Introduction

The eye is an immune privileged site where the introduction of antigens does not elicit an inflammatory immune response [1]. Several strategies are used to protect the eye from pathogens. The first line of protection is the blood-ocular barrier, which impedes harmful pathogens from entering the eye via the peripheral bloodstream [2]. A regional immune system provides a second multilayered defense in case the blood-ocular barrier is breached. The anterior chamber is bathed in aqueous humor fluid, which is strongly immunosuppressive and profoundly inhibits T cell activation [1]. In addition, low expression of major histocompatibility complex (MHC) class II molecules limits antigen presentation to immune cells reducing the chances of an immune response. Stromal cells of the iris and ciliary body have the ability to convert effector T cells to regulatory T cells and expression of death-inducing molecules results in apoptosis of immune cells keeping the immune response in check [3]. However, ocular inflammation can still occur in spite of these multiple overlapping mechanisms. Furthermore, aqueous humor outflow regulation, which is important in glaucoma, may be impacted by the innate immune system [4].

## 2. Glaucoma

**Figure 1:**
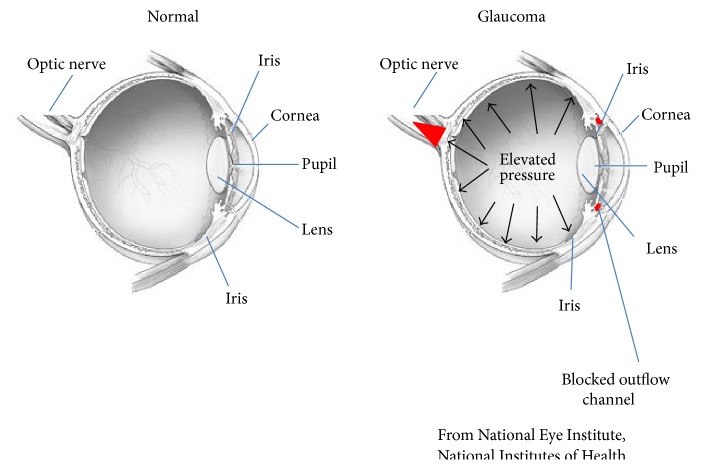
Schematic showing the anatomy of normal and glaucoma eyes. In glaucoma, a blockage in the aqueous humor outflow pathway in the anterior chamber (red) causes intraocular pressure to increase leading to loss of retinal ganglion cells and optic nerve damage at the back of the eye (orange). Images were slightly modified from those freely provided by the National Eye Institute, National Institute of Health.

The glaucomas are a group of optic neuropathies with a characteristic pattern of damage to the optic nerve that leads to loss of peripheral vision (see Figure 1) [5]. A leading cause of global irreversible blindness, glaucoma will impact 111.8 million individuals by 2040 [6]. The number of affected individuals is likely to be much higher than the reported number because the disease is usually asymptomatic up until major neural damage has occurred [7, 8]. Primary open angle glaucoma (POAG) is the most common form of glaucoma [6].

The exact etiology of POAG remains unknown. It is clear that it is a heterogeneous group of disorders with multiple causative factors including gene mutations, environmental factors, and certain medications. Moreover, there are several risk factors that predispose to the disease including advanced age, elevated intraocular pressure, race, and family history [9–12]. While lowering intraocular pressure often reduces the rate of vision loss, many patients continue to go blind despite apparently “successful” pressure control [10, 11, 13]. Since there is no single causative factor, there are potentially many disease mechanisms behind glaucoma. Some lead to higher intraocular pressures, while others affect how the optic nerve withstands either pressure fluctuations or sustained elevation of intraocular pressure. Thus, glaucoma research has focused on the front of the eye, where intraocular pressure is regulated by a tissue called the trabecular meshwork, the back of the eye, where studies have focused on retinal ganglion cell physiology and optic nerve damage, and distal changes in retinal ganglion cell axons in terminal projection sites such as the superior colliculus and the lateral geniculate [14–18].

Diagnostic criteria for glaucoma have been revamped significantly over the past 40 years with increased emphasis on characteristic changes in the optic disc and retinal nerve fiber layer and less reliance on elevated intraocular pressure [9, 19]. Normal tension glaucoma, that is, patients with statistically normal intraocular pressures, makes up 30 to 40% of glaucoma cases [13, 20]. Even for these patients, the only effective treatment for glaucoma continues to be reduction of intraocular pressure levels by either pharmaceutical or surgical means.

**Figure 2:**
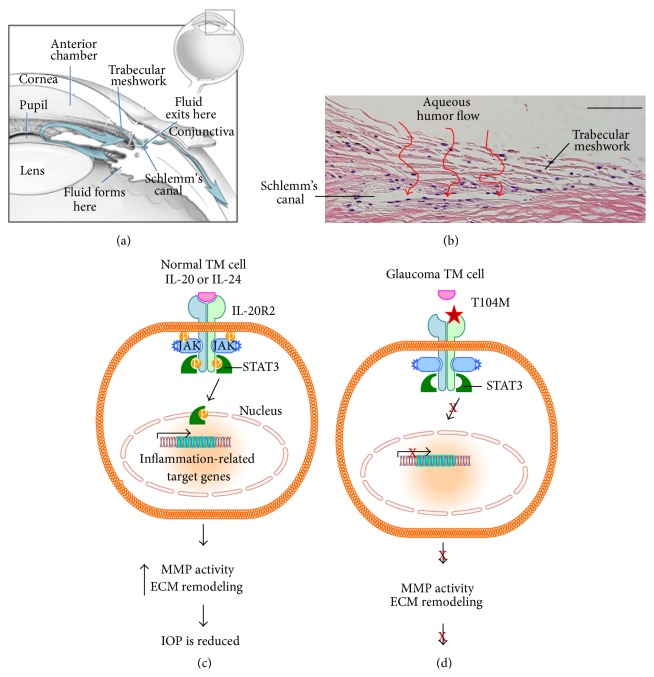
(a) Schematic of the anterior chamber of the eye showing the location of the trabecular meshwork and the flow pattern of aqueous humor. Image was slightly modified from those freely provided by the National Eye Institute, National Institute of Health. (b) H&E stained radial section of a human trabecular meshwork. The TM is a triangular-shaped tissue comprised of a series of fenestrated beams around which the aqueous humor flows (red arrows) before draining into Schlemm's canal. For orientation purposes, the cornea is to the right and the ciliary body is toward the left. (c) Schematic of IL-20 signaling in normal cells. IL-20 or IL-24 binds to the IL-20RB receptor, which phosphorylates Janus kinase (JAK). JAK then phosphorylates STAT3, which translocates to the nucleus to promote transcription of inflammation-related target genes. This in turn increases MMP activity and ECM remodeling. (d) In glaucoma cells harboring the* IL-20RB T104M* mutation, the cytokine is unable to bind to the receptor so the JAK/STAT3 pathway is not activated. Therefore, higher expression of proinflammatory genes remains and elevated IOP would be sustained since MMP activity and ECM remodeling are not affected.

Physiological intraocular pressures are established by maintaining a balance between production and drainage of aqueous humor in the anterior chamber. Aqueous humor is continuously produced by the ciliary processes and it bathes tissues in the anterior chamber before exiting out to Schlemm's canal via a filter-like tissue called the trabecular meshwork (see Figure 2). Building resistance to aqueous humor outflow in the trabecular meshwork produces a tunable system by which this filter can increase or decrease outflow when needed [21]. Dysfunction of this conventional outflow pathway leads to impaired drainage and elevated intraocular pressure, as is seen in POAG patients [14]. Increased intraocular pressure places excessive mechanical stresses on the lamina cribrosa of the optic nerve in the posterior segment of the eye and loss of retinal ganglion cells ensues. Once lost, these retinal ganglion cells cannot be regenerated. Since these cells are responsible for transmitting visual signals to the brain, irreversible blindness occurs [22].

Inflammatory responses may contribute to the glaucomatous process as shown by studies in humans and rodent models [23–26]. In the anterior segment, certain inflammatory cytokines have altered expression levels in the aqueous humor of glaucomatous eyes compared to age-matched normal eyes. These include interleukin-6 (IL-6), transforming growth factor beta-1 (TGF*β*1), TGF*β*2, IL-6, IL-8, IL-10, IL-12, *α*-serum amyloid A, interferon-*γ* (IFN*γ*), and CXL9 [27–33]. The source of these cytokines in the aqueous humor of glaucoma patients is not clear. However, acute elevation of intraocular pressure, such as in primary angle-closure glaucoma, damages the blood-aqueous-barrier (BAB), which can lead to leakage of the cytokines into the aqueous humor [34]. Inflammation-related changes also occur in the posterior segment. In a mouse model of laser-induced ocular hypertension, there was upregulated expression of MHC-II and glial fibrillary acidic protein (GFAP) in the microglia of contralateral eyes. The authors suggest that microglial activation in the nontreated eye could be related to an immune response [35, 36]. In humans, however, a systemic autoimmune response is less convincing because the contralateral eye in patients with unilateral glaucoma does not appear to exhibit glaucomatous degenerative changes [37]. However, proinflammatory cytokines such as tumor necrosis factor-*α* (TNF*α*) and its receptor are upregulated in glaucomatous human optic nerve [38–40]. Moreover, use of bupropion, which suppresses TNF*α* production, significantly lowered the risk of developing POAG in humans in a large retrospective study [41], while anti-TNF*α* medication (etanercept) was found to be neuroprotective in a rodent model of glaucoma [42]. Thus, both in glaucoma patients and in rodent models of glaucoma, there is accumulating evidence for a potential role of the immune system in contributing to deleterious changes in anterior and posterior ocular tissues. Our recent identification of a mutation in* interleukin-20 receptor-B* (*IL-20RB*) supports this contention [43].

## 3. IL-20RB Mutation Associated with POAG

Our group mapped a gene in a large POAG Oregon family to chromosome 3, the* GLC1C* locus [44]. Eighty-six family members, ranging in age from 8 to 91 years old, had extensive ophthalmic examinations. Thirteen family members were diagnosed with POAG and twelve of these had elevated intraocular pressure (22 to 49 mm Hg). An additional nine individuals, who do not have POAG at this time, had elevated intraocular pressures. After refining the region to 4 cM [45], we sequenced all 49 genes in the* GLC1C* locus and identified one nonsynonymous mutation: a T104M change in* IL-20RB* (rs367923973) [43]. This is an extremely rare variant with a reported frequency of 0.02% in dbSNP (http://www.ncbi.nlm.nih.gov/SNP). This mutation, T104M, lies in IL-20RB's active binding site for the cytokines, IL-19, IL-20, and IL-24, which are all members of the IL-20 subfamily of interleukins (see below) [46]. The T104 site in IL-20RB binds to S111 in IL-20 [46]. Substitution of T104 with a methionine would replace the hydroxyl group that forms a hydrogen bond with S111 in IL-20 with a sulfate group, thus disrupting the bond between the cytokine and its receptors [46]. Based on these findings, this* IL-20RB* mutation is highly likely to impact the IL-20 signaling pathway, which may contribute to the pathogenesis of glaucoma in this family.

## 4. The IL-20 Family of Cytokines and Receptors

The IL-20 subfamily of cytokines and receptors are members of the larger IL-10 family, which are grouped together based on their utilization of common receptor subunits, similarities in their target-cell profiles, and biological functions [47]. This subfamily consists of the cytokines, IL-19, IL-20, IL-22, IL-24, and IL-26, as well as the receptors, IL-20RA, IL-20RB, IL-10RB, and IL-22RA1 [47]. IL-19 exclusively signals through the IL-20RA/IL-20RB heterodimer, while IL-20 and IL-24 can use both the IL-20RA/IL-20RB heterodimer and the IL-22RA1/IL-20RB receptor. IL-22 signals through IL-22RA and IL-10RB, while IL-26 uses IL-20RA and IL-10RB [47]. It should be noted that IL-20RA and IL-20RB are also known as IL-20R1 and IL-20R2, respectively. The IL-20 subfamily participates both in amplifying inflammatory responses particularly during autoimmune and chronic inflammation and alternatively in anti-inflammatory responses, such as tissue protection and regeneration [47, 48]. Thus, understanding how regulation of this subfamily is occurring is paramount in devising new treatment strategies for patients in this large POAG family.

Both IL-20RA and IL-20RB are expressed in normal human trabecular meshwork cell lysates and are upregulated in response to cytokine treatment [43].* IL-20RB* mRNA is also expressed in moderately high levels in both the retinal ganglion cell layer and the optic nerve head in rats (Dr. Elaine Johnson, personal communication). Human aqueous humor contains IL-20, IL-24, IL-20RA, and IL-20RB protein [49]. IL-20, IL-24, IL-20RA, and IL-20 RB are expressed in the retina and optic nerve head in the DBA/2J mouse model of glaucoma (see later) [50]. However, IL-19 is not expressed in the retina or optic nerve head of DBA/2J mice [50].

## 5. Downstream Signaling via IL-20RB

Binding of IL-20 to its receptor activates the Janus kinase- (JAK-) signal transducer and activator of transcription (STAT) pathway (JAK-STAT) [51, 52]. Elevation of intraocular pressure has also been shown to activate the JAK-STAT pathway [53], which is involved in retinal ganglion cell survival [54]. Activation of STAT3 can be both pro- and anti-inflammatory even within the same cell type, but how the desired response is elicited remains a question [55]. For example, IL-6 and IL-10 both activate STAT3, but they generate different cellular responses with IL-6 generating a proinflammatory response and IL-10 producing an anti-inflammatory one [55]. These differences appear to correlate with the level of STAT3 over time, with IL-6 producing a transient activation while IL-10 generates a sustained level of STAT3 activation [55]. The STAT3-induced protein, suppressor of cytokine signaling-3 (SOCS3), may be involved because it mediates signaling dynamics due to its ability to inhibit signals from the IL-6 receptor, but not the IL-10 receptor [55].

STAT3 signaling can influence matrix metalloproteinase (MMP) activity. MMPs are a family of enzymes involved in remodeling of extracellular microenvironments [56]. Several studies have linked IL-20 signaling to MMP levels and activity. In breast cancer cells, IL-20 upregulates MMP-9 and MMP-12 [57]. Furthermore, MMP-3 levels are increased by IL-20 in cultured human invertebral disc cells [58]. IL-20 appears to act synergistically with IL-1*β* in these cells: Higher levels of TNF*α*, IL-1*β*, IL-6, IL-8, MMP-3, and monocyte chemoattractant protein-1 (MCP1) were found when disc cells were treated with both cytokines compared to exposure to IL-20 or IL-1*β* alone [58]. MMP activity has been implicated in regulation of intraocular pressure [59–63]. Induction of MMP activity decreases the resistance to aqueous humor outflow through the trabecular meshwork which, in turn, lowers intraocular pressure [61].

## 6. Functional Consequences of the* IL-20RB* Mutation in POAG Fibroblasts

**Figure 3:**
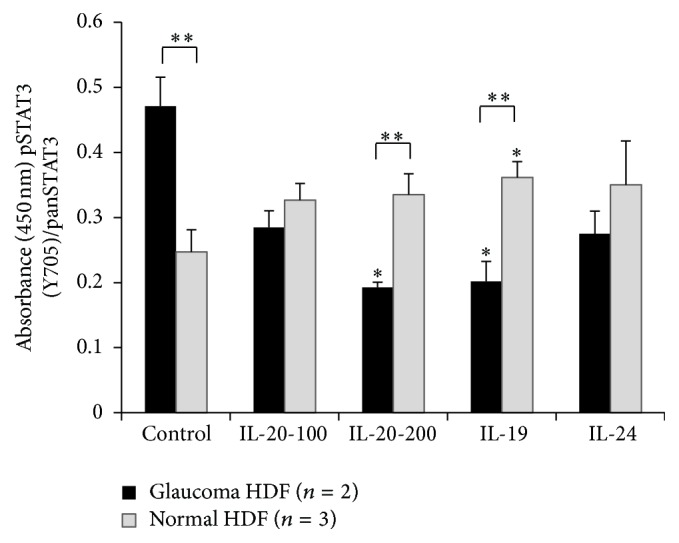
STAT3 activation in normal human dermal fibroblasts (white bars, *n* = 3) and patient fibroblasts with the* IL-20RB T104M* mutation (dark bars, *n* = 2) with and without cytokine stimulation: ^*∗*^ *p* < 0.05 between cytokine treated and untreated cells; ^*∗∗*^ *p* < 0.01 and ^*∗∗∗*^ *p* < 0.05 comparing fibroblasts with the* T104M IL-20RB* mutation versus wild-type cells with the same cytokine treatment (reprinted with copyright permission from Journal of Ocular Pharmacology and Therapeutics). IL-20 was added at 100 or 200 ng/mL and IL-19 and IL-24 were used at 100 ng/mL.

**Figure 4:**
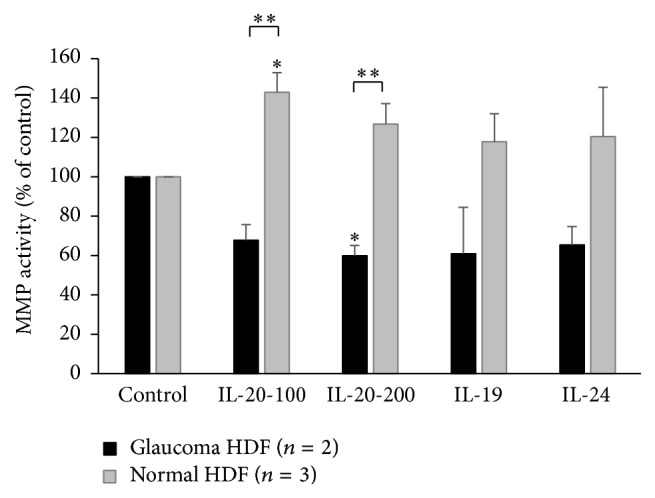
MMP activity in conditioned media from normal (white bars, *n* = 3) and patient fibroblasts with the* IL-20RB T104M* mutation (dark bars, *n* = 2) treated with cytokines ^*∗*^ *p* < 0.03 (reprinted with copyright permission from Journal of Ocular Pharmacology and Therapeutics). IL-20 was added at 100 or 200 ng/mL and IL-19 and IL-24 were used at 100 ng/mL.

To investigate whether normal downstream signaling pathways were active in mutant cells, we asked if IL-20 treatment induces STAT3 phosphorylation and MMP activity in normal dermal fibroblasts and in POAG patient fibroblasts with the* IL-20RB* mutation. Fibroblasts from glaucoma patients with the* T104M IL-20RB* mutation had higher basal levels of phosphorylated STAT3 compared to wild-type fibroblasts (see Figure 3) [43]. Stimulation of wild-type fibroblasts by IL-19, IL-20, or IL-24 cytokines led to a significant increase in phosphorylation of STAT3 after 15 minutes, but this was not found in glaucomatous fibroblasts with the* T104M IL-20RB* mutation [43]. Using a quenched fluorescent peptide assay that produces fluorescent signal when cleaved by a MMP, we showed that IL-20 increased MMP activity in wild-type human fibroblasts, but not in fibroblasts with the* T104M IL-20RB* mutation (see Figure 4). The differential response of mutant and wild-type cells to cytokine treatment suggested that POAG cells with the* T104M* mutation are unable to launch an appropriate cell signaling response upon stimulation by the IL-20 family of cytokines.

Collectively, these observations suggest that, in normal cells, IL-20 and related cytokines bind to IL-20 receptors on the cell surface (Figure 2(c)). This would in turn activate STAT3, which would translocate to the nucleus to modify transcription of inflammatory-related genes [64]. The anti-inflammatory response would include upregulation of IL-20 receptors by trabecular meshwork cells and activation of MMPs leading to remodeling of the extracellular matrix. This would lead, ultimately, to greater outflow of aqueous humor [59]. In glaucoma patients with the* IL-20RB* mutation (Figure 2(d)), the IL-20 family of cytokines would not bind efficiently to the IL-20RA/RB receptor so STAT3 would not be activated and translocated to the nucleus. Therefore, the anti-inflammatory response would not be adequate and sustained expression of proinflammatory genes would remain. Subsequently, many secondary downstream effects may contribute to the elevated intraocular pressure observed in glaucoma patients harboring the* IL-20RB* mutation and, ultimately, to glaucomatous damage [43].

## 7. The IL-20 Family and the DBA/2J Mouse Model of Glaucoma

The DBA/2J mouse model of glaucoma is an inbred mouse strain that progressively develops glaucoma-like abnormalities with aging. In the anterior chamber, the mice develop a form of pigment dispersion syndrome with the primary action being an inflammatory response resulting in elevation of intraocular pressure [65]. The DBA/2J mouse has two distinct phenotypes: iris pigment dispersion, which may be involved in immune dysfunction in DBA/2J eyes, and iris stromal atrophy. These phenotypes are caused by mutations in the* Gpnmb* and* Tyrp1* genes, respectively, [66, 67].* Gpnmb* is expressed in some types of dendritic cells [68, 69], which are potent professional antigen presenting cells, whereas* Tyrp1* is an antigen that is involved in inflammatory eye disease [70]. As discussed above, normal aqueous humor inhibits T cell activation and is strongly immunosuppressive. However, the aqueous humor of DBA/2J mice lacks immunosuppressive properties and the capacity to support anterior chamber associated immune deviation [65].

The DBA/2J mouse develops glaucoma secondary to its pigment disease. Iris pigment is shed into the aqueous humor, where it enters the outflow pathways and eventually causes blockage of the drainage channels. This causes progressive elevation of pressure [71]. Following elevation of intraocular pressure, DBA/2J mice between 6 and 7 months of age begin to lose retinal ganglion cells. By 10–12 months, significant retinal ganglion cell loss has occurred in the majority of DBA/2J mice [72, 73]. However, if the mice are exposed to a high dose of *γ*-irradiation, the DBA/2J mice are protected from developing glaucomatous damage, although they still have high intraocular pressures [73, 74]. Both retinal and optic nerve morphology appear normal in the irradiated mice, whereas untreated DBA/2J mice show optic nerve atrophy, as well as clear loss of retinal ganglion cell axons [73].

A unique finding in the DBA/2J mice is a high level of activated microglia in the inner central retina and optic nerve region at 3 months, which occurs well before the loss of retinal ganglion cells [75]. IL-19 is the mostly highly upregulated gene in activated microglia and, in addition, the IL-20RA and IL-20RB receptors are expressed [76]. IL-19 has anti-inflammatory activity in microglia via STAT3 activation, which leads to an anti-inflammatory response [76]. Irradiation reduces the number of proliferating microglia in the optic nerve head along with a reduction in the levels of microglia activation in the central retina, optic nerve head, and laminar region in the DBA/2J mouse [74, 77]. Minocycline, a neuroprotective tetracycline derivative that suppresses chronic neuroinflammation and microglial activation, also has a protective effect on retinal ganglion cell viability in the DBA/2J mouse [78].

In DBA/2J mice with moderate axon damage, IL-24 expression is significantly increased in the retina, but no significant differences were seen for either IL-20 or IL-20RB [50]. Conversely, IL-20RA levels are significantly reduced in optic nerves from eyes with severe axon damage in DBA/2J mice compared to DBA/2J-*Gpnmb* ^+^ [50]. The reduction of IL-20RA levels as axon damage becomes severe suggests that heterodimeric IL-20RA/IL-20RB signaling is impaired, which could ultimately lead to altered extracellular matrix remodeling by MMPs via inappropriate STAT3 activation as described above. Changes in extracellular matrix composition and organization in the glaucomatous optic nerve head have been well documented and include deposition of extracellular matrix materials in areas formerly occupied by axons [79]. The extracellular matrix changes likely contribute to the altered biomechanical properties of the glaucomatous optic nerve head and increase the vulnerability of the remaining axons to cell death [80, 81].

## 8. Conclusions

In conclusion, several lines of evidence provide compelling evidence for a role of the IL-20 family of cytokines in glaucoma. First, we have identified a mutation in IL-20RB at a residue that is critical for binding of the receptor to the IL-20 family of cytokines in a large POAG pedigree. Second, IL-20 stimulation of the mutant IL-20RB leads to abnormal STAT3 activation and MMP activity in glaucoma fibroblasts. Third, IL-20 family members and their receptors have altered expression levels in the retina and optic nerve head of the DBA/2J mouse, which develops glaucoma-like symptoms with aging. While the* IL-20RB* mutation most likely is not a causative factor in the majority of glaucoma cases, the identification of this mutation has revealed that defective IL-20 signaling may lead to glaucoma in this large POAG pedigree. Future studies will focus on the role of the IL-20 subfamily members in both aqueous outflow regulation in normal trabecular meshwork and their anti-inflammatory role in the posterior tissues of the eye. This may lead to the development of novel therapeutic treatments to maintain tissue homeostasis and prevent glaucomatous vision loss in this large Oregon POAG family.
